# Exploring Co-Occurring Clinical and Treatment Characteristics Associated with In Vitro Fertilization Failure in Polycystic Ovary Syndrome: An Association Rule Mining Approach

**DOI:** 10.3390/healthcare14162508

**Published:** 2026-08-12

**Authors:** Xuehong Zhu, Lina Ge, Guanghui Dong, Yanlin Tang, Zhong Lin, Feng Han

**Affiliations:** 1 The Reproductive Hospital of Guangxi, No. 3 Longyuan Road, Qingxiu District, Nanning 530006, China; 202510135@sr.gxmu.edu.cn (X.Z.); 1155011427@link.cuhk.edu.hk (Z.L.); 2 School of Artificial Intelligence, Guangxi University for Nationalities, No. 188 Daxue East Road, Nanning 530006, China; 19920028@gxmzu.edu.cn (L.G.); yanlintang@stu.gxmzu.edu.cn (Y.T.); 3 Guangxi Key Laboratory of Hybrid Computation and IC Design Analysis, No. 188 Daxue East Road, Nanning 530006, China; 4 School of Public Health, Sun Yat-sen University, No. 74 Zhongshan Second Road, Guangzhou 510080, China; donggh5@mail.sysu.edu.cn

**Keywords:** association rule mining, in vitro fertilization, polycystic ovary syndrome, retrospective cohort, clinical pregnancy failure, co-occurrence patterns

## Abstract

**Background:** Polycystic ovary syndrome (PCOS) is a leading cause of anovulatory infertility and a frequent indication for in vitro fertilization/intracytoplasmic sperm injection (IVF/ICSI). Multivariable regression estimates adjusted associations, whereas association rule mining (ARM) can describe frequently co-occurring attributes; however, ARM outputs are unadjusted and may be sensitive to analytic specification. **Objectives:** We used ARM alongside multivariable logistic regression and internal robustness analyses to describe combinations of clinical and treatment-related variables that co-occurred with clinical pregnancy failure among women with PCOS undergoing IVF/ICSI. Methods: We retrospectively analyzed 733 deidentified patients with PCOS from the Reproductive Hospital of Guangxi. The original one-hot-encoded analytic file was linked to a supplemental clinical record file containing continuous age, BMI, infertility duration, anti-Müllerian hormone, total gonadotropin dose, fertilization method, embryo-transfer day, number of embryos transferred, and embryo score. Embryo quality was derived from D3 cleavage-stage and blastocyst morphology records. Multivariable logistic regression was used to estimate adjusted associations. ARM results were assessed using Fisher’s exact test, Benjamini–Hochberg false discovery rate (FDR) correction, 1000 bootstrap resamples, fivefold cross-validation, and threshold/cutoff sensitivity analyses. **Results:** In the multivariable model (n = 730), age remained associated with clinical pregnancy failure (adjusted odds ratio [aOR] = 1.04 per year, 95% CI 1.00–1.08, *p* = 0.031), whereas BMI was not independently associated with failure (aOR = 1.00 per kg/m^2^, 95% CI 0.96–1.05, *p* = 0.860). Transfer of two embryos (aOR = 0.55, 95% CI 0.35–0.85, *p* = 0.008), D5 blastocyst transfer (aOR = 0.49, 95% CI 0.32–0.75, *p* = 0.001), and transfer of at least one high-quality embryo (aOR = 0.63, 95% CI 0.43–0.93, *p* = 0.020) were associated with lower odds of failure. Under the primary host-factor specification, age > 35 years + BMI ≥ 24 kg/m^2^ + pelvic adhesion defined 48 patients, 30 of whom had clinical pregnancy failure (support = 0.07; confidence = 0.63; lift = 1.38; FDR q = 0.098). In the secondary treatment-inclusive analysis, infertility duration > 5 years + no high-quality embryo transferred defined 43 patients with 30 failures (support = 0.06; confidence = 0.70; lift = 1.54; FDR q = 0.026). Lift represents the subgroup failure proportion divided by the overall failure proportion and is not an adjusted risk ratio. The rule composition and the number of FDR-supported rules changed under alternative cutoffs and confidence thresholds. **Conclusions:** ARM described transparent but specification-sensitive co-occurrence profiles that complement, rather than compete with, regression. Treatment-inclusive profiles combine baseline and downstream cycle characteristics and are therefore especially exploratory. These findings require prospective multicenter validation before any clinical decision-support use.

## 1. Introduction

Polycystic ovary syndrome (PCOS) is a common endocrine and metabolic disorder that affects women of reproductive age [1] and is characterized by hyperandrogenism, ovulatory dysfunction, and polycystic ovarian morphology. PCOS affects approximately 5–20% of women worldwide and is the leading cause of anovulatory infertility [2]. While lifestyle modifications and ovulation induction enable many PCOS patients to conceive, a substantial proportion ultimately require assisted reproductive technologies (ARTs) such as in vitro fertilization (IVF).

When women with PCOS undergo IVF, they face distinct clinical challenges. Although controlled ovarian stimulation (COS) in PCOS often yields a large cohort of oocytes, many of the oocytes are of suboptimal quality and result in poor-quality embryos [3]; COS also increases the risk of ovarian hyperstimulation syndrome. Consequently, patients with PCOS tend to exhibit lower implantation and clinical pregnancy rates per cycle. Despite advances in IVF techniques and a deeper understanding of the pathophysiology of PCOS, many patients still fail to conceive following treatment.

A variety of patient factors influence IVF outcomes among PCOS patients. Advanced maternal age [4], obesity (high body mass index [BMI]) [1,5], prolonged infertility [6,7], hormonal imbalances [8,9], tubal disease [10,11], and uterine abnormalities have all been linked to poor outcomes [12]. In particular, metabolic and hormonal disturbances inherent to PCOS (such as insulin resistance and hyperandrogenism) can synergistically impair reproduction. For example, elevated androgen levels combined with insulin resistance have been demonstrated to negatively affect oocyte development and endometrial receptivity. A recent review [13] highlighted these pathophysiologic mechanisms as resulting in abnormal follicle growth, poor oocyte maturation, and a dysfunctional endometrial lining—all of which contribute to infertility in patients with PCOS.

Because these factors interact in complex ways, conventional analyses may not directly summarize the combinations of attributes that occur within clinically recognizable subgroups. Machine-learning methods can accommodate numerous variables and nonlinear structure, and previous studies have shown that data-driven models using age, BMI, and other clinical features can support individualized prediction of IVF outcomes [14,15,16].

Association rule mining (ARM) constitutes a powerful data mining technique for uncovering hidden “if–then” patterns across multiple variables and has been widely deployed in healthcare to identify interpretable relationships among clinical factors [17]. For example, association rules have been adopted to construct diagnostic knowledge bases and to summarize how combinations of patient characteristics jointly influence outcomes [18]. This is in contrast to previous studies in which risk factors for clinical pregnancy outcomes were often assessed independently, while the multifactorial interactions that frequently influenced these outcomes were overlooked.

We therefore applied ARM to clinical data from women with PCOS undergoing IVF/ICSI to describe combinations of characteristics that co-occurred with clinical pregnancy failure. Multivariable regression and ARM were used to answer different questions: regression estimated adjusted associations between measured predictors and failure, whereas ARM summarized unadjusted co-occurrence patterns. The two approaches were not treated as alternative prediction models, and all ARM profiles were considered specification-dependent hypotheses requiring external validation.

## 2. Methods and Materials

### 2.1. Data Sources

We conducted a single-center retrospective cohort study using deidentified electronic medical records (EMRs) from the Reproductive Hospital of Guangxi. The final analytic cohort contained 733 unique patients with PCOS, each represented by one eligible IVF/ICSI cycle. The original binary analytic file was matched one-to-one with a supplemental clinical record file by iCI_ID/CI ID. The Supplemental File provided continuous age, BMI, infertility duration, anti-Müllerian hormone, basal endocrine indicators, total gonadotropin dose, fertilization method, embryo-transfer day, number of embryos transferred, and embryo score. All 733 records were matched exactly once; no duplicate matched identifiers or missing clinical pregnancy outcomes were found. The Supplemental File did not contain a distinct field for the number of oocytes retrieved or the detailed ovarian stimulation protocol.

Clinical treatment context: The analyzable records captured conventional IVF versus ICSI; total gonadotropin dose; embryo transfer on D3, D5, or occasionally D6; transfer of one or two embryos; and morphology-based embryo scores. Detailed controlled ovarian stimulation regimens, trigger strategies, endometrial preparation protocols, and oocyte counts were not retained in the analytic extract; therefore, protocol-specific distributions and effects could not be estimated.

### 2.2. Data Preprocessing

The raw data from the EMRs were rigorously preprocessed to ensure high-quality inputs for analysis, with the following steps implemented.

(1) Terminology standardization: Clinical descriptions and diagnoses were normalized to a consistent vocabulary. Identical concepts appeared under different terms or abbreviations in the raw records. For example, “polycystic ovary syndrome” was recorded as “polycystic ovarian syndrome”, “PCOS”, or “poly-ovary syndrome”. Synonyms and abbreviations were harmonized to a single descriptor so that each clinical concept was represented consistently across records.
(2) Record filtering, de-duplication, and missing-data handling: The analysis file was checked for duplicate patient/cycle identifiers and missing values before statistical modeling. Duplicate detection was performed using the iCI_ID, and no duplicate records were found (n = 0). Complete-case checking across the 32 original analytic columns found no missing cells (n = 0); therefore, no additional records were removed during the present re-analysis. The final cohort contained 733 records. Because the uploaded file represented the cleaned analytic extract, source-level counts before EMR export (e.g., total registrations screened and exclusions before deidentification) were verified against the hospital extraction log if required for the final submission.
(3) Continuous variables, one-hot encoding, and treatment-variable derivation: The original analytic file contained one-hot-encoded clinical items, whereas the Supplemental File provided continuous age, BMI, infertility duration, gonadotropin dose, anti-Müllerian hormone, and embryo-transfer variables. Age and BMI were analyzed continuously in multivariable regression. For ARM and sensitivity analyses, the primary cutoffs were age > 35 years, BMI ≥ 24 kg/m^2^ (the Chinese adult overweight threshold), and infertility duration > 5 years; alternative cutoffs were examined in sensitivity analyses. D3 embryos were classified as high quality when they were grade 1–2 with 7–9 cells. Blastocysts were classified as high quality when expansion was ≥3 and both inner-cell-mass and trophectoderm grades were A or B. Ambiguous or compacted notations were conservatively classified as not meeting the high-quality definition unless another transferred embryo in the same record met the criteria.

These preprocessing steps produced a standardized dataset suitable for regression and exploratory ARM.

### 2.3. Feature Selection

We extracted all clinically relevant variables available in the cleaned analytic and Supplemental Files and constructed two prespecified itemsets, presented in Table 1. The primary host-factor set included age, BMI, infertility duration, infertility type, uterine abnormalities, tubal factors, pelvic adhesion, LUFS, and endocrine/metabolic comorbidities. A secondary treatment-inclusive set additionally included fertilization method, gonadotropin dose, number of embryos transferred, embryo-transfer day, and derived embryo-quality indicators. This secondary set was used to examine how downstream cycle characteristics altered observed co-occurrence patterns; it was not intended to control for confounding or estimate treatment effects. Oocyte count and detailed stimulation protocol were unavailable and were treated as unmeasured covariates.

All variables used for ARM were converted into binary (0/1) items following preprocessing. The complete item dictionary, including item names, source EMR fields, coding rules, missing-data handling, and clinical rationales, has been added as Supplementary Table S1. Feature selection was guided by clinical expertise and the prior literature on PCOS and infertility. Treatment-related variables available in the EMRs were included where consistently recorded; variables not reliably available in the current database, such as embryo ploidy status, detailed embryo morphology, some sperm parameters, and endometrial preparation details, were added to the limitations as potential unmeasured confounders.

### 2.4. ARM

ARM is an unadjusted, data-driven pattern-discovery technique for identifying interpretable “if–then” co-occurrence patterns among discrete items [27]. Apriori was used to discover frequent itemsets, and rules were then constrained to those with clinical pregnancy failure as the consequent [28,29]. The resulting analysis is outcome-constrained exploratory rule mining; it does not estimate adjusted effects, establish causality, or identify which antecedent item drives the observed outcome proportion.

The primary thresholds were prespecified as support ≥ 0.05, confidence ≥ 0.50, lift > 1.00, and antecedent length ≤ 3. The primary host-factor ARM excluded post-transfer variables. The secondary treatment-inclusive ARM added treatment and embryo-transfer variables to assess how cycle context changed the descriptive rule set. Because these secondary rules combine baseline patient characteristics with downstream treatment decisions and embryo features, they were interpreted as hybrid co-occurrence profiles rather than patient phenotypes or treatment effects.

Frequent itemset generation: The one-hot encoded matrix was used to identify frequent itemsets. For an itemset X, support was calculated as the proportion of records containing X:

Support(X) = n(X)/N.(1)

Itemsets with support ≥ 0.05 were retained in the primary analysis. ARM was repeated at support thresholds of 0.03, 0.05, and 0.07 to quantify sensitivity to the minimum subgroup-frequency requirement.

Association rule extraction: Rules were generated from frequent itemsets using the mlxtend.frequent_patterns.apriori and association_rules functions. For a rule X → Y, confidence was defined as the conditional probability of Y among records containing X:

Confidence(X → Y) = n(X ∩ Y)/n(X).(2)

Rules with confidence ≥ 0.50 and lift > 1.00 were retained. Lift was calculated as the ratio of the observed confidence to the marginal probability of the consequent:

Lift(X → Y) = Confidence(X → Y)/P(Y).(3)

For the outcome-constrained rule X → clinical pregnancy failure, lift equals P(failure|X)/P(failure). Thus, a lift of 1.38 means that the observed failure proportion in records containing X was 1.38 times the overall cohort failure proportion. It does not mean a 38% adjusted or causal increase in risk. Values close to 1.00 indicate limited descriptive enrichment; values around or above 1.50 indicate larger enrichment in the observed dataset, but no universal lift threshold establishes clinical importance. Lift must be interpreted together with subgroup size, confidence intervals, FDR results, and sensitivity analyses.

Rule filtering and statistical assessment: In the host-factor ARM, 78 support-eligible antecedent combinations were evaluated; 38 rules met support ≥ 0.05, confidence ≥ 0.50, and lift > 1.00, and 32 nonredundant rules remained after pruning supersets that did not change support or confidence. In the secondary treatment-inclusive ARM, 242 candidate combinations were evaluated; 141 met the primary filters, and 121 nonredundant rules remained after pruning. Fisher’s exact test was applied to each candidate rule, followed by Benjamini–Hochberg FDR correction; q < 0.10 was used as an exploratory retention criterion.

### 2.5. Multivariable Regression and ARM Robustness Assessment

Multivariable logistic regression estimated adjusted cohort-level associations between measured predictors and clinical pregnancy failure. The model included continuous age, BMI, infertility duration, infertility type, uterine abnormalities, tubal factors, pelvic adhesion, LUFS, endocrine/metabolic disorders, fertilization method, gonadotropin dose, number of embryos transferred, embryo-transfer day, and embryo-quality status. A categorical sensitivity model used age > 35 years, BMI ≥ 24 kg/m^2^, and infertility duration > 5 years. ARM stability was examined using 1000 bootstrap resamples and fivefold stratified cross-validation. Regression and ARM were not compared as alternative risk models: regression estimated adjusted associations, whereas ARM described unadjusted combinations and observed subgroup proportions. No discrimination, calibration, decision-curve, or prospective decision-impact comparison was performed.

### 2.6. Computational Environment

We conducted all analyses using Python 3.12 in a Jupyter Notebook v 7.0 environment. Statistical testing used SciPy 1.16.3 and statsmodels 0.14.5; logistic regression used statsmodels Logit; and ARM rule enumeration, Fisher’s exact testing, FDR correction, bootstrap resampling, and 5-fold validation were implemented in Python, as shown in Table 2. Data summaries and supplementary output tables were exported as Excel files.

**Figure 1:**
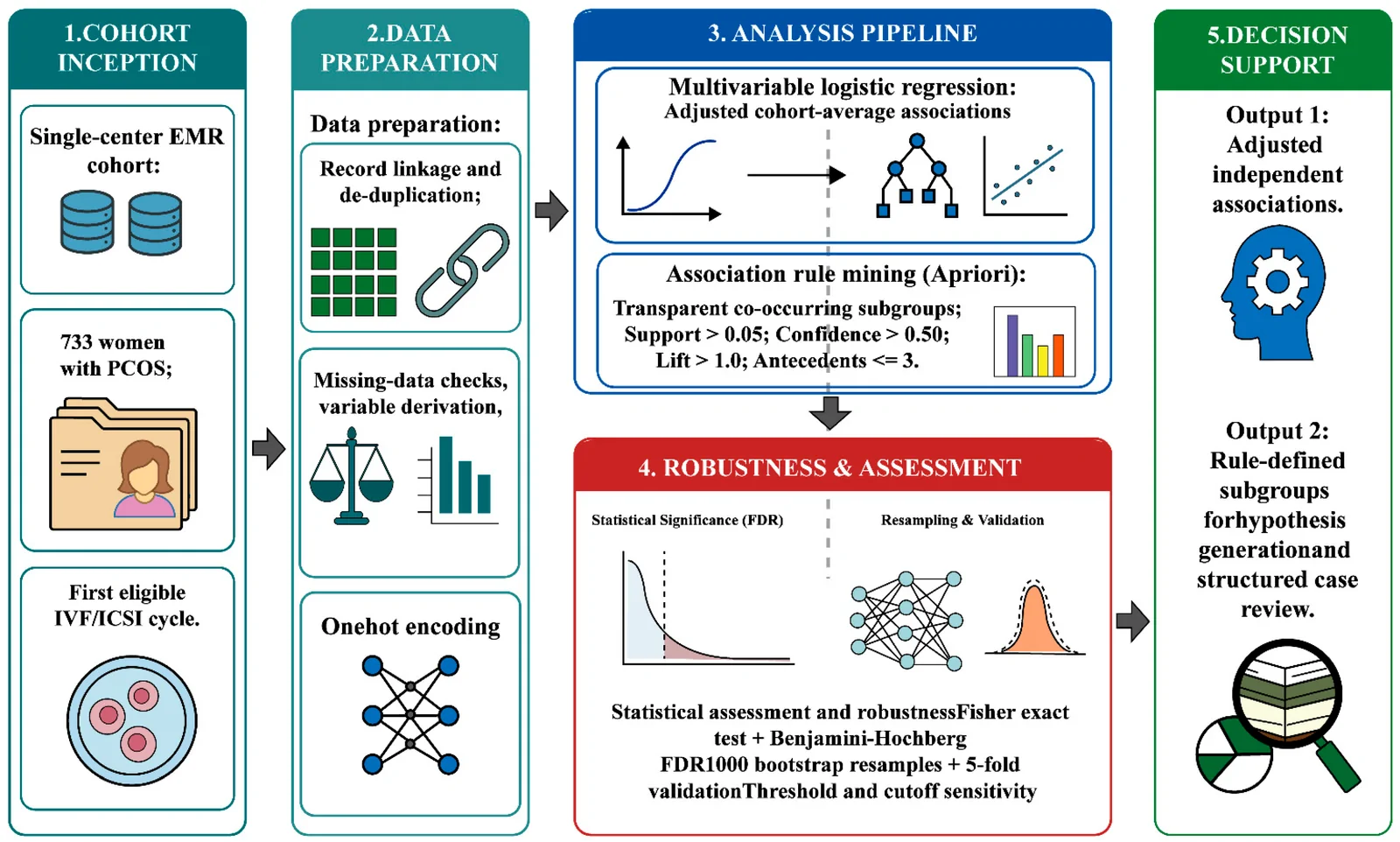
Complementary analytical framework.

**Figure 2:**
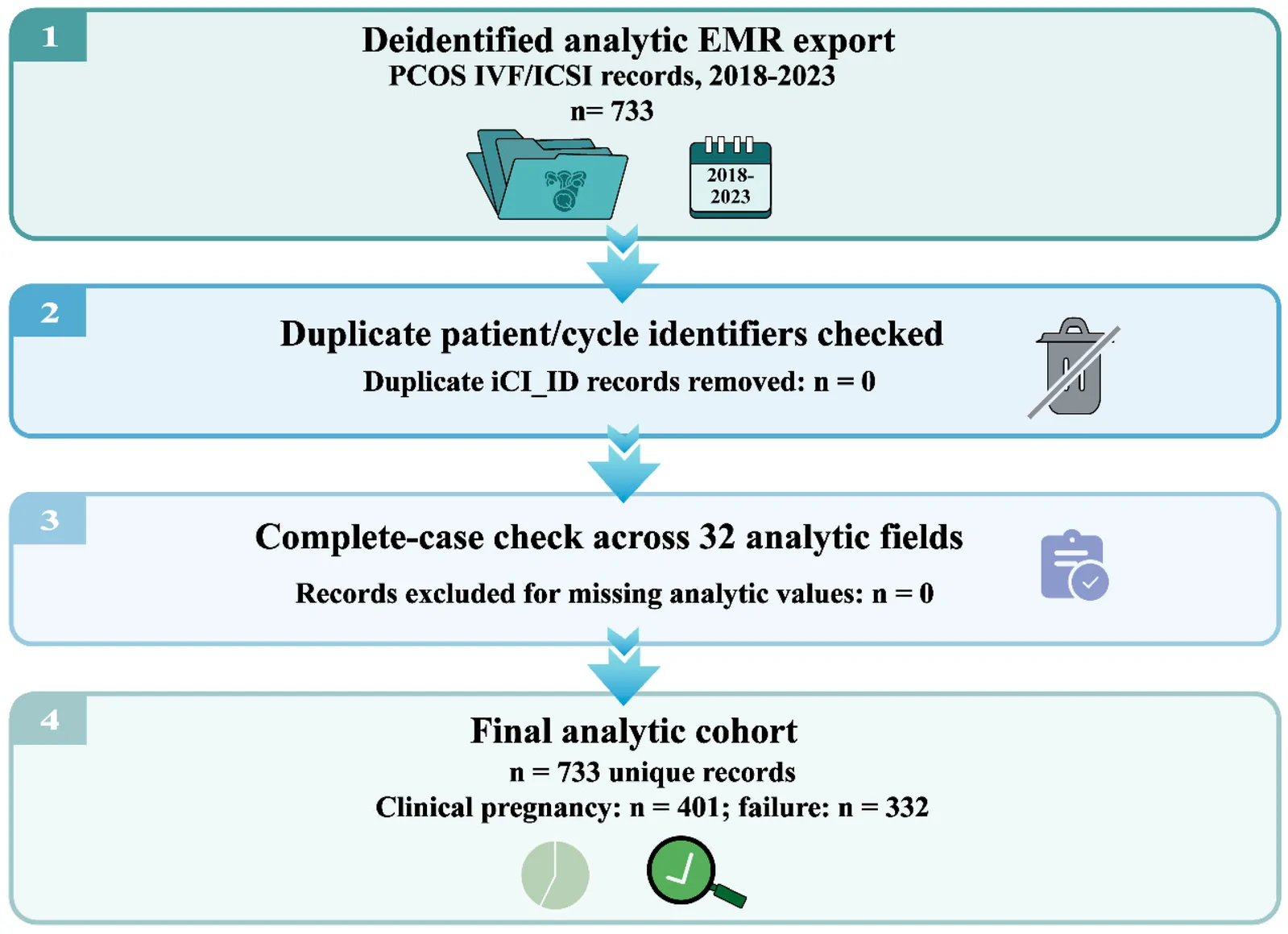
Patient selection and formation of the final analytic cohort (n = 733).

Figure 1 summarizes the complementary analytical workflow, and Figure 2 shows the formation of the final analytic cohort.

## 3. Results

### 3.1. Demographic and Clinical Characteristics

After the original analytic file was matched to the supplemental clinical records, the final analysis included 733 unique patients with PCOS. Clinical pregnancy occurred in 401 patients (54.7%), whereas 332 patients (45.3%) experienced clinical pregnancy failure. Continuous age, BMI, infertility duration, gonadotropin dose, number of embryos transferred, embryo-transfer day, and embryo-quality variables were available, except for three missing infertility-duration values and 68 missing anti-Müllerian hormone values. Oocyte count was not available.

**Figure 3:**
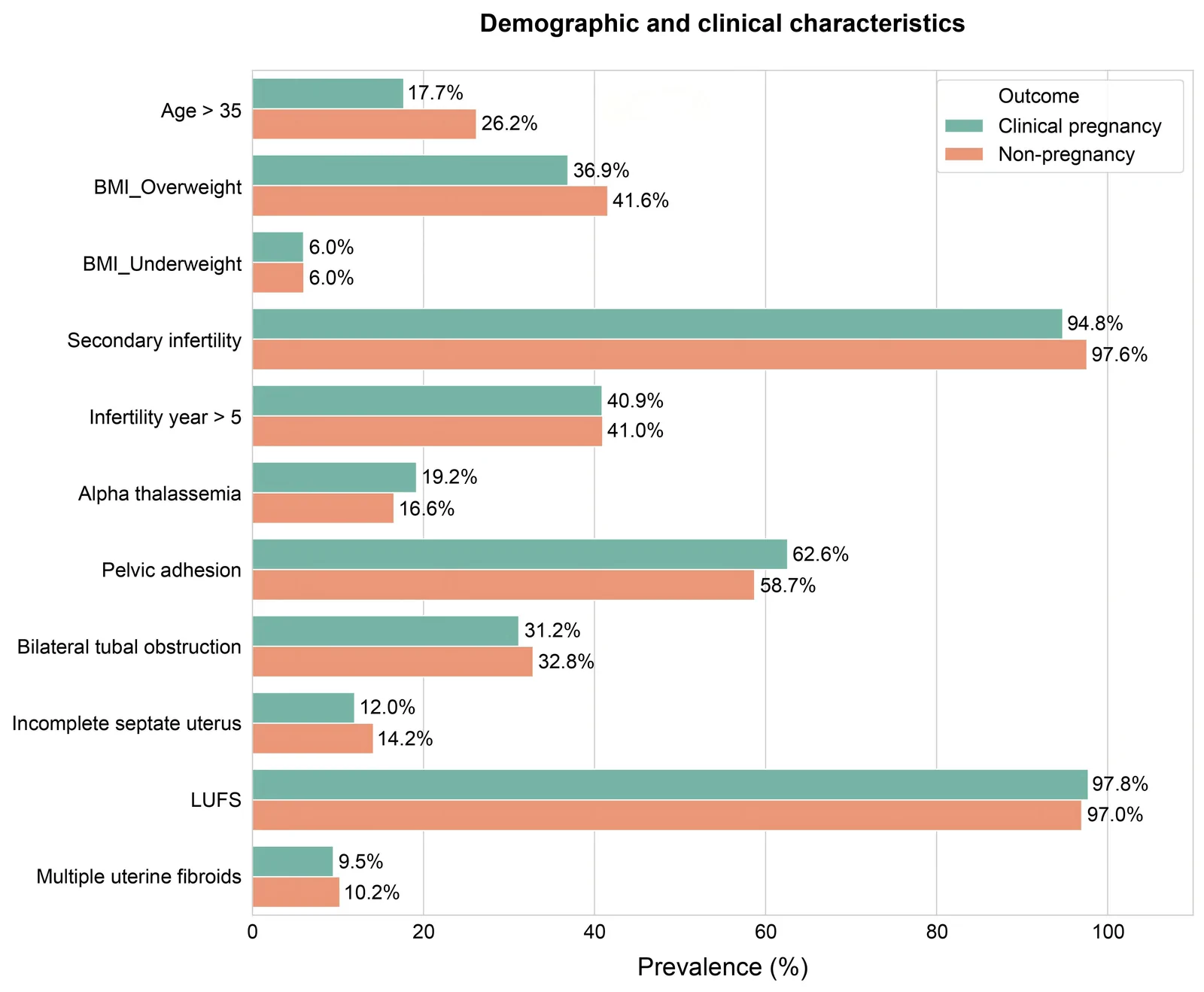
Distribution of predefined categorical host factors by clinical pregnancy outcome.

The predefined categorical host-factor items were illustrated in Figure 3: Age >35 years was more frequent in the clinical pregnancy failure group than in the clinical pregnancy group (26.2% versus 17.7%, *p* = 0.007), whereas continuous age did not differ significantly (32.09 ± 5.02 versus 31.39 ± 4.31 years, *p* = 0.100). BMI and infertility duration also did not differ significantly. ICSI was more frequent in the failure group (20.8% versus 15.2%, *p* = 0.049), and transfer of at least one high-quality embryo was less frequent (78.0% versus 84.5%, *p* = 0.023).

These unadjusted comparisons do not account for correlated host and treatment characteristics. Multivariable regression was therefore used to estimate adjusted associations, whereas ARM was used only to describe combination-based subgroups.

### 3.2. Multivariable Logistic Regression Results

In the complete-case multivariable model (n = 730), presented in Table 3, age remained associated with clinical pregnancy failure (aOR = 1.04 per year, 95% CI 1.00–1.08, *p* = 0.031), whereas BMI was not independently associated with failure (aOR = 1.00 per kg/m^2^, 95% CI 0.96–1.05, *p* = 0.860). Secondary infertility showed a nonsignificant positive association (aOR = 2.30, 95% CI 0.95–5.61, *p* = 0.066). ICSI was associated with higher odds of failure (aOR = 1.56, 95% CI 1.02–2.38, *p* = 0.039). Transfer of two embryos (aOR = 0.55, 95% CI 0.35–0.85, *p* = 0.008), D5 blastocyst transfer (aOR = 0.49, 95% CI 0.32–0.75, *p* = 0.001), and transfer of at least one high-quality embryo (aOR = 0.63, 95% CI 0.43–0.93, *p* = 0.020) were associated with lower odds of failure.

### 3.3. Illustrative Association-Rule Findings Under the Primary Specification

At the prespecified thresholds, the primary host-factor ARM yielded 32 nonredundant rules, seven with FDR q < 0.10. One illustrative retained profile was age > 35 years + BMI ≥ 24 kg/m^2^ + pelvic adhesion (n = 48; failures = 30; support = 0.07; confidence = 0.63; lift = 1.38; FDR q = 0.098). In the secondary treatment-inclusive ARM, 121 nonredundant rules were retained, 11 with FDR q < 0.10. An illustrative treatment-inclusive profile was infertility duration > 5 years + no high-quality embryo transferred (n = 43; failures = 30; support = 0.06; confidence = 0.70; lift = 1.54; FDR q = 0.026). These profiles were selected for concise presentation because they had comparatively large observed lift values under the primary specification; this selection does not imply a hierarchy of clinical importance. Complete rule lists, bootstrap intervals, and fivefold validation statistics are provided in Supplementary Tables S2 and S3.

**Figure 4:**
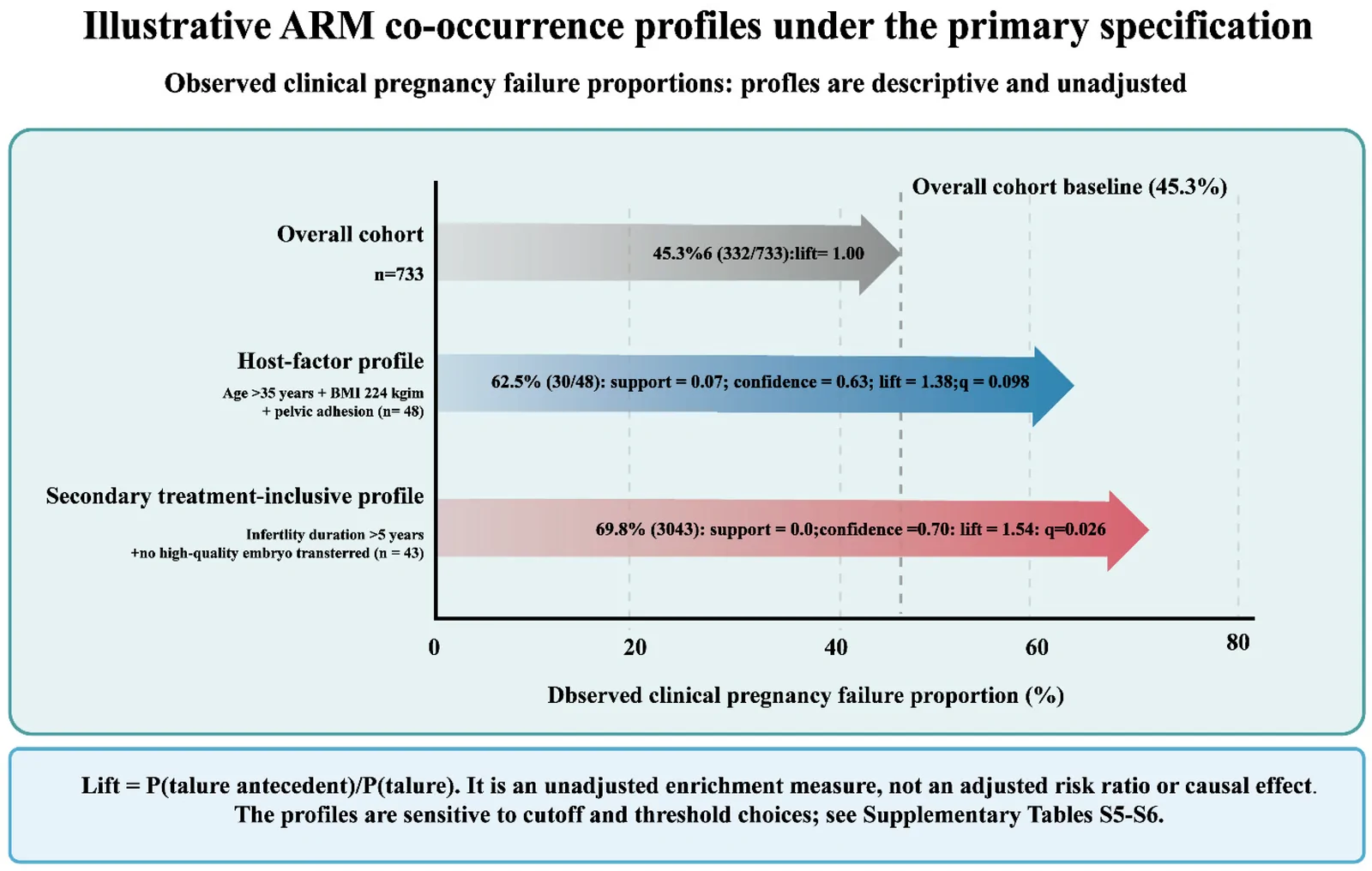
Illustrative ARM co-occurrence profiles under the primary specification.

The bars in Figure 4 show observed clinical pregnancy failure proportions in the overall cohort and two rule-defined subgroups selected for concise presentation. Lift is P(failure|antecedent)/P(failure); it is an unadjusted enrichment measure, not an adjusted risk ratio. Selection by observed lift does not imply clinical priority, causality, or treatment benefit. The profiles are sensitive to cutoff and threshold choices (Supplementary Tables S5 and S6).

### 3.4. Sensitivity and Secondary ARM Findings

The sensitivity analyses demonstrated substantial specification dependence. At support = 0.05, increasing the confidence threshold from 0.50 to 0.60 reduced the number of FDR-supported host-factor rules from seven to three and the number of FDR-supported treatment-inclusive rules from 11 to seven. Changing the support threshold also changed the rule with the largest observed lift. When the age cutoff was increased to >38 years, the age–BMI–pelvic-adhesion profile was no longer retained as an FDR-supported rule; at >40 years, no host-factor rule met FDR q < 0.10. Raising the BMI cutoff from 24 to 25 or 28 kg/m^2^ also changed the composition of the illustrative profile. These findings indicate that the ARM outputs are not robust to modest analytic changes and should not be treated as stable clinical indicators. Detailed sensitivity results are provided in Supplementary Tables S5 and S6.

## 4. Discussion

Regression and ARM provided complementary but fundamentally different views of the data. Regression estimated adjusted average associations for measured predictors. ARM described unadjusted combinations and observed outcome proportions without controlling for other variables. The ARM outputs therefore cannot identify which item within a combination drives the observed enrichment, and they should not be interpreted as adjusted risk estimates or rankings of clinical importance. The sensitivity analyses further showed that the retained rule set depended materially on discretization and threshold choices.

### 4.1. Principal Findings and Relation to Conventional Regression

Age remained associated with failure after multivariable adjustment, whereas BMI did not. The co-occurrence of age and BMI in a host-factor profile therefore does not establish an independent BMI effect or a biological interaction; it only identifies a subgroup in which those attributes were jointly present. Similarly, treatment-inclusive profiles showed larger observed lift values in some specifications, but these unadjusted values do not demonstrate stronger causal or clinical effects.

The two methods should not be compared as competing risk models. Regression addresses the adjusted association of each measured predictor with the outcome, conditional on the other modeled variables. ARM addresses the descriptive question of which discrete attributes occur together and what outcome proportion was observed in those records. For ARM, lift values only slightly above 1.00 indicate modest enrichment relative to the cohort baseline. Values around or above 1.50 indicate larger descriptive enrichment in the analyzed dataset, but no universal lift threshold establishes clinical meaningfulness. Interpretation must account for subgroup size, uncertainty, multiple testing, and specification sensitivity.

### 4.2. Current Clinical Interpretability and Potential Future Use

The present rules are not sufficiently stable or externally validated for clinical decision support. At most, after replication across centers and analytic specifications, a rule-matching display could be evaluated as a secondary structured-review prompt alongside established clinical assessment and adjusted evidence. It might direct attention to co-occurring anatomic, metabolic, cycle, or embryo characteristics during case review and counseling [30,31]. Rule membership must not automatically trigger weight-loss treatment, surgery, embryo-number changes, or other interventions [32,33]. Prospective decision-impact studies are required to determine whether displaying validated profiles changes decisions or improves outcomes beyond usual care.

### 4.3. Interpretation of the Secondary Treatment-Inclusive ARM

The treatment-inclusive ARM was a secondary and more exploratory analysis because it combined baseline patient characteristics with downstream treatment decisions and embryo features. For example, the profile “infertility duration > 5 years + no high-quality embryo transferred” cannot determine whether the observed failure proportion reflects infertility duration, embryo quality, their correlation, laboratory grading practices, or other unmeasured cycle factors. Embryo quality may also lie on a pathway between patient characteristics and outcome [33]. Adding these variables to ARM does not adjust for confounding or estimate mediation; it only shows that the descriptive rule set changes when cycle-level information is included. These hybrid profiles should therefore not be interpreted as patient phenotypes or treatment effects.

### 4.4. Unmeasured Confounding and Generalizability

Treatment and embryo variables were prominent in both regression and descriptive ARM, making unavailable covariates especially important. Detailed stimulation regimen and oocyte number may influence ovarian response, embryo availability, transfer-day selection, and embryo number. Embryo ploidy may explain implantation failure not captured by morphology; sperm parameters may affect fertilization and embryo development; and endometrial preparation may affect receptivity [34,35]. Their omission creates residual confounding and possible confounding by indication because clinicians select protocols and transfer strategies in response to patient and cycle characteristics. Available variables—total gonadotropin dose, fertilization method, transfer day, embryo number, and derived embryo quality—reduced the information gap but did not resolve it.

External validity is limited by the single-center Chinese setting. The analyzable treatment context included conventional IVF/ICSI, D3/D5/occasional D6 transfer, and one- or two-embryo transfer, but detailed stimulation and endometrial-preparation protocols were unavailable. Other populations and IVF programs may differ in PCOS phenotype distribution, BMI definitions, referral patterns, stimulation regimens, ICSI use, embryo culture and grading, transfer policies, and access to genetic testing [36]. Consequently, the observed support, confidence, lift, and rule composition should not be assumed to transport to other ethnic groups, laboratories, or healthcare systems. Multicenter external validation should re-estimate all rule metrics under local definitions and protocols [37,38].

### 4.5. Strengths and Limitations

Strengths include the linked clinical dataset, prespecified primary thresholds, adjusted regression, multiple-testing correction, bootstrap intervals, fivefold internal validation, and explicit cutoff/threshold sensitivity analyses. The limitations are substantial: the retrospective single-center design, modest subgroup sizes, discretization of continuous variables, residual and indication-related confounding, and absence of external or prospective decision-impact validation. Most importantly, moderate changes in age, BMI, support, and confidence thresholds materially changed the rule composition and FDR-supported counts; at an age cutoff of >40 years, no host-factor rule met q < 0.10. Internal resampling does not overcome this specification fragility or establish transportability. The ARM results should therefore remain exploratory until replicated across populations, protocols, and analytic definitions.

## 5. Conclusions

This study used multivariable regression and ARM to address different questions in a single-center PCOS-IVF/ICSI cohort. Regression estimated adjusted cohort-level associations, whereas ARM described transparent but unadjusted co-occurrence profiles. The illustrative host-factor and treatment-inclusive profiles had higher observed failure proportions than the cohort baseline, but ARM could not determine which antecedent item drove those proportions or whether any relationship was causal.

The sensitivity analyses showed that moderate changes in age, BMI, support, and confidence thresholds materially altered the rule composition and the number of FDR-supported rules. Treatment-inclusive profiles also mixed baseline characteristics with downstream cycle and embryo variables. Accordingly, these findings are specification-sensitive hypotheses, not stable or generalizable clinical indicators, and they should not be used for decision support or treatment selection. Prospective multicenter validation with complete protocol, oocyte, ploidy, sperm, and endometrial data, followed by formal assessment of calibration, net benefit, and decision impact, is required before any clinical implementation.

## Figures and Tables

**Table 1:** Features extracted from EMRs.

Domain	Extracted Variables	References
**Demographics**	Female age (continuous); primary ARM cutoff > 35 years; alternative cutoffs assessed in sensitivity analyses	[19,20,21]
**Anthropometry**	BMI (continuous); primary ARM cutoff ≥ 24 kg/m^2^; alternative cutoffs ≥ 25 and ≥28 kg/m^2^ assessed in sensitivity analyses	
**Reproductive history**	Infertility duration (continuous); primary ARM cutoff > 5 years; alternative cutoffs > 7 and >10 years; infertility type, primary/secondary	[22,23]
**Hormonal and metabolic disorders**	Hyperprolactinemia, subclinical hypothyroidism, insulin resistance	[5,8,9,24]
**Tubo-uterotubal factors**	Unilateral tubal obstruction, bilateral tubal obstruction, hydrosalpinx, pelvic adhesion, intrauterine adhesion	[11,25]
**Ovarian and endocrine factors**	Luteinized unruptured follicle syndrome (LUFS), ovarian cysts	[11,25]
**Uterine malformations**	Incomplete septate uterus and multiple uterine fibroids	[12]
**ART treatment data**	Fertilization method (conventional IVF versus ICSI), total gonadotropin dose, embryo-transfer day (D3, D5, or D6), number of embryos transferred (one or two), and derived high-quality-embryo status	[26]
**Outcome**	Clinical pregnancy, 1 = success, 0 = failure	

BMI, body mass index; ART, assisted reproductive technology; IVF, in vitro fertilization; ICSI, intracytoplasmic sperm injection; EMR, electronic medical record.

**Table 2:** Computational environment for this study.

Category	Specification	Purpose
**Programming language**	Python 3.12	Core scripting/analysis
**Interactive IDE**	Jupyter Notebook v7.0.0	Reproducible, stepwise workflow
**Key libraries**	Pandas 2.3.1, NumPy 2.2.3, SciPy 1.16.3, statsmodels 0.14.5, MLxtend 0.23.4, matplotlib 3.10.3	Data handling, logistic regression, ARM, validation, and visualization
**Plotting**	matplotlib, seaborn	Descriptive and network visualization
**Hardware**	2 × Intel Xeon (48 physical cores), 128 GB RAM	Parallel support-counting and rule filtering
**Operating system**	Ubuntu 22.04 LTS	Stable Linux environment for multithreaded tasks

IDE, integrated development environment; RAM, random access memory; LTS, long-term support.

**Table 3:** Multivariable logistic regression model for clinical pregnancy failure.

Variable	Adjusted OR	95% CI	*p*-Value	Note
**Age, per year**	1.04	1.00–1.08	0.031	Primary continuous predictor
BMI, per kg/m^2^	1.00	0.96–1.05	0.860	Continuous BMI
**Infertility duration, per year**	0.96	0.92–1.01	0.140	
**Secondary infertility**	2.30	0.95–5.61	0.066	
**Incomplete septate uterus**	0.97	0.61–1.55	0.895	
**Multiple uterine fibroids**	0.93	0.56–1.57	0.798	
**Bilateral tubal obstruction**	1.13	0.80–1.62	0.484	
**Pelvic adhesion**	0.95	0.67–1.33	0.759	
**LUFS**	0.63	0.25–1.61	0.334	
**Insulin resistance**	1.38	0.53–3.60	0.510	
**Subclinical hypothyroidism**	1.20	0.51–2.82	0.675	
ICSI versus conventional IVF	1.56	1.02–2.38	0.039	
Gonadotropin dose, per 1000 IU	1.00	0.81–1.24	0.998	
Two embryos transferred versus one	0.55	0.35–0.85	0.008	
D5 blastocyst transfer versus D3/D6	0.49	0.32–0.75	0.001	Treatment/transfer variable
**At least one high-quality embryo transferred**	0.63	0.43–0.93	0.020	Derived from embryo score

## Data Availability

The datasets used and/or analyzed during the current study are available from the corresponding author upon reasonable request, subject to approval by the Ethics Committee of the Reproductive Hospital of Guangxi and compliance with applicable data protection regulations. Requests for data access should be directed to the corresponding author (Feng Han, fenghan@gxmzu.edu.cn) and will be evaluated on a case-by-case basis to ensure ethical and legal compliance.

## Supplementary Materials

The following supporting information can be downloaded at: https://www.mdpi.com/article/10.3390/healthcare14162508/s1. Table S1, Complete ARM item dictionary; Table S2, Host-factor ARM rules with patient counts, support, confidence, lift, Fisher *p*-values, Benjamini–Hochberg FDR q-values, bootstrap confidence intervals, and 5-fold validation results; Table S3, Full clinical/treatment ARM rules; Table S4, Multivariable logistic regression; Table S5, Support/confidence threshold sensitivity; Table S6, Age/BMI/infertility duration cutoff sensitivity.

## Abbreviations

The following abbreviations are used in this manuscript:

ART	assisted reproductive technology
BMI	body mass index
COS	controlled ovarian stimulation
EMR	electronic medical record
ICSI	intracytoplasmic sperm injection
IDE	integrated development environment
IVF	in vitro fertilization
LTS	long-term support
LUFS	luteinized unruptured follicle syndrome
PCOS	polycystic ovary syndrome
RAM	random access memory
