# Supplementary material for: Exploring Co-Occurring Clinical and Treatment Characteristics Associated with In Vitro Fertilization Failure in Polycystic Ovary Syndrome: An Association Rule Mining Approach
Source: Healthcare (Basel). 2026 Aug 12;14(16):2508. doi: 10.3390/healthcare14162508 (PMC13512646; doi:10.3390/healthcare14162508)
Supplement: Supplementary file 1 [file healthcare-14-02508-s001.zip › healthcare-4374784-supplementary.pdf]

Table S1. Complete ARM item dictionary.

Item	Domain	Operational definition used for ARM	Source/derivation
Age >35 years	Demographics	Maternal age categorized as >35 years; alternative cutoffs of 38 and 40 years were tested in sensitivity analysis.	Derived from continuous age in supplemental clinical records
BMI ≥24 kg/m²	Anthropometry/metabolic	BMI categorized using the Chinese adult overweight threshold of ≥24 kg/m²; alternative cutoffs of 25 and 28 kg/m² were tested.	Derived from continuous BMI
Infertility duration >5 years	Reproductive history	Infertility duration longer than 5 years; alternative cutoffs of >7 and >10 years were tested.	Derived from continuous years of infertility
Secondary infertility	Reproductive history	Secondary infertility recorded in the EMR/clinical dataset.	Extracted from EMR
Pelvic adhesion	Tubo-uterine factor	Pelvic adhesion recorded as a tubo-uterine/anatomic comorbidity.	Extracted from EMR diagnosis fields
Bilateral tubal obstruction	Tubo-uterine factor	Bilateral tubal obstruction recorded as a tubal-factor diagnosis.	Extracted from EMR diagnosis fields
Incomplete septate uterus	Uterine malformation	Incomplete septate uterus recorded as a uterine anomaly.	Extracted from EMR diagnosis fields
Multiple uterine fibroids	Uterine factor	Multiple uterine fibroids/leiomyomas recorded in the EMR.	Extracted from EMR diagnosis fields
LUFS	Ovarian/endocrine factor	Luteinized unruptured follicle syndrome.	Extracted from EMR diagnosis fields
Insulin resistance	Endocrine/metabolic factor	Insulin resistance recorded in the clinical dataset.	Extracted from EMR/laboratory diagnosis fields
Subclinical hypothyroidism	Endocrine/metabolic factor	Subclinical hypothyroidism recorded in the clinical dataset.	Extracted from EMR/laboratory diagnosis fields
ICSI	Treatment/fertilization	Intracytoplasmic sperm injection rather than conventional IVF.	Derived from fertilization method
Gn dose ≥ median	Treatment/stimulation	Gonadotropin dose greater than or equal to the cohort median of 1575 IU.	Derived from Gn dose
Single embryo transferred	Embryo transfer	One embryo transferred in the analyzed cycle.	Derived from number of embryos transferred
Day-3/6 embryo transfer	Embryo transfer	Embryo transfer day/type other than D5 blastocyst transfer.	Derived from embryo transfer type
No high-quality embryo transferred	Embryo quality	No transferred embryo met the study definition of good-quality embryo.	Derived from embryo score/grade
Clinical pregnancy failure	Outcome	Absence of clinical pregnancy, defined as no fetal heartbeat on ultrasound at approximately 6–7 weeks after embryo transfer.	Derived from clinical pregnancy outcome

Table S2. Host-factor ARM rules with patient counts, support, confidence, lift, Fisher p-values, Benjamini–Hochberg FDR q-values, bootstrap confidence intervals, and 5-fold validation results.

Rule	Antecedent	n antecedent	n failure	Support	Confidence	Lift	Fisher p	BH-FDR q	Bootstrap 95% CI: confidence	Bootstrap 95% CI: lift	5-fold CV confidence	5-fold CV lift
1	Age >35 years + BMI ≥24 kg/m² + Pelvic adhesion	48	30	0.065	0.625	1.38	0.010	0.098	0.487–0.750	1.10–1.66	0.602 ± 0.149	1.33 ± 0.33
2	Age >35 years + Incomplete septate uterus	37	23	0.050	0.622	1.37	0.026	0.155	0.467–0.774	1.02–1.72	0.619 ± 0.158	1.37 ± 0.34
3	Age >35 years + BMI ≥24 kg/m² + Secondary infertility	88	54	0.120	0.614	1.35	<0.001	0.053	0.516–0.714	1.14–1.57	0.613 ± 0.027	1.35 ± 0.06
4	Age >35 years + BMI ≥24 kg/m² + Infertility duration >5 years	44	27	0.060	0.614	1.35	0.020	0.155	0.465–0.750	1.03–1.66	0.582 ± 0.155	1.28 ± 0.34
5	Age >35 years + BMI ≥24 kg/m²	90	54	0.123	0.600	1.32	0.002	0.053	0.500–0.700	1.11–1.53	0.599 ± 0.037	1.32 ± 0.08
6	Age >35 years + Infertility duration >5 years + Pelvic adhesion	46	26	0.063	0.565	1.25	0.077	0.215	0.429–0.698	0.95–1.54	0.527 ± 0.121	1.16 ± 0.26
7	BMI ≥24 kg/m² + Infertility duration >5 years + Pelvic adhesion	70	39	0.095	0.557	1.23	0.043	0.161	0.444–0.672	0.99–1.47	0.555 ± 0.146	1.22 ± 0.32
8	Age >35 years + Secondary infertility	155	86	0.211	0.555	1.22	0.003	0.054	0.483–0.626	1.08–1.37	0.560 ± 0.066	1.24 ± 0.15
9	Age >35 years + Infertility duration >5 years	74	41	0.101	0.554	1.22	0.043	0.161	0.442–0.658	0.98–1.46	0.559 ± 0.055	1.23 ± 0.13
10	Age >35 years	158	87	0.216	0.551	1.22	0.004	0.056	0.478–0.621	1.07–1.36	0.555 ± 0.061	1.23 ± 0.14
11	Age >35 years + Secondary infertility + Pelvic adhesion	89	49	0.121	0.551	1.22	0.032	0.155	0.453–0.648	1.01–1.42	0.554 ± 0.022	1.22 ± 0.05
12	Age >35 years + Pelvic adhesion	91	50	0.124	0.549	1.21	0.031	0.155	0.456–0.644	1.01–1.41	0.552 ± 0.028	1.22 ± 0.06
13	Age >35 years + Secondary infertility + LUFS	153	84	0.209	0.549	1.21	0.005	0.063	0.476–0.621	1.06–1.36	0.555 ± 0.060	1.22 ± 0.13

Rule	Antecedent	n antecedent	n failure	Support	Confidence	Lift	Fisher p	BH-FDR q	Bootstrap 95% CI: confidence	Bootstrap 95% CI: lift	5-fold CV confidence	5-fold CV lift
14	Age >35 years + Infertility duration >5 years + LUFS	73	40	0.100	0.548	1.21	0.056	0.181	0.435–0.657	0.97–1.44	0.550 ± 0.050	1.21 ± 0.11
15	BMI ≥24 kg/m² + Pelvic adhesion + Bilateral tubal obstruction	44	24	0.060	0.545	1.20	0.132	0.272	0.400–0.698	0.90–1.55	0.542 ± 0.261	1.20 ± 0.58
16	Age >35 years + LUFS	156	85	0.213	0.545	1.20	0.006	0.068	0.472–0.617	1.05–1.35	0.549 ± 0.055	1.21 ± 0.12
17	Infertility duration >5 years + Bilateral tubal obstruction + LUFS	68	37	0.093	0.544	1.20	0.073	0.210	0.422–0.655	0.95–1.45	0.561 ± 0.096	1.24 ± 0.21
18	Age >35 years + Secondary infertility + Bilateral tubal obstruction	57	31	0.078	0.544	1.20	0.097	0.245	0.410–0.667	0.93–1.47	0.557 ± 0.108	1.23 ± 0.24
19	Age >35 years + Pelvic adhesion + LUFS	89	48	0.121	0.539	1.19	0.051	0.181	0.440–0.632	0.99–1.39	0.539 ± 0.029	1.19 ± 0.07
20	BMI ≥24 kg/m² + Secondary infertility + Incomplete septate uterus	56	30	0.076	0.536	1.18	0.124	0.262	0.403–0.667	0.89–1.48	0.545 ± 0.051	1.20 ± 0.11
21	Age >35 years + Bilateral tubal obstruction	58	31	0.079	0.534	1.18	0.123	0.262	0.406–0.657	0.92–1.45	0.548 ± 0.116	1.21 ± 0.26
22	BMI ≥24 kg/m² + Incomplete septate uterus	58	31	0.079	0.534	1.18	0.123	0.262	0.397–0.662	0.91–1.45	0.538 ± 0.023	1.19 ± 0.05
23	Infertility duration >5 years + Bilateral tubal obstruction	70	37	0.095	0.529	1.17	0.113	0.262	0.413–0.639	0.92–1.42	0.537 ± 0.060	1.19 ± 0.13
24	BMI ≥24 kg/m² + Infertility duration >5 years + LUFS	114	60	0.156	0.526	1.16	0.054	0.181	0.436–0.620	0.98–1.34	0.534 ± 0.098	1.18 ± 0.21
25	Age >35 years + Bilateral tubal obstruction + LUFS	57	30	0.078	0.526	1.16	0.154	0.305	0.396–0.653	0.89–1.43	0.541 ± 0.110	1.19 ± 0.24
26	BMI ≥24 kg/m² + Infertility duration >5 years	117	61	0.160	0.521	1.15	0.064	0.193	0.430–0.610	0.97–1.33	0.527 ± 0.079	1.16 ± 0.17
27	Secondary infertility + Pelvic adhesion + Incomplete septate uterus	50	26	0.068	0.520	1.15	0.200	0.325	0.375–0.660	0.84–1.46	0.517 ± 0.082	1.14 ± 0.18
28	Pelvic adhesion + Incomplete septate uterus	52	27	0.071	0.519	1.15	0.197	0.325	0.385–0.656	0.85–1.45	0.517 ± 0.088	1.14 ± 0.19

Rule	Antecedent	n antecedent	n failure	Support	Confidence	Lift	Fisher p	BH-FDR q	Bootstrap 95% CI: confidence	Bootstrap 95% CI: lift	5-fold CV confidence	5-fold CV lift
29	BMI ≥24 kg/m² + Infertility duration >5 years + Bilateral tubal obstruction	43	22	0.059	0.512	1.13	0.261	0.348	0.364–0.667	0.81–1.46	0.539 ± 0.109	1.19 ± 0.24
30	Pelvic adhesion + Incomplete septate uterus + LUFS	51	26	0.070	0.510	1.13	0.241	0.348	0.373–0.648	0.84–1.43	0.511 ± 0.080	1.13 ± 0.17
31	BMI ≥24 kg/m² + Secondary infertility + LUFS	307	154	0.419	0.502	1.11	0.015	0.129	0.446–0.553	1.02–1.21	0.501 ± 0.039	1.11 ± 0.08

Table S3. Full clinical/treatment ARM rules.

Rule	Antecedent	n antecedent	n failure	Support	Confidence	Lift	Fisher p	BH-FDR q	Bootstrap 95% CI: confidence	Bootstrap 95% CI: lift	5-fold CV confidence	5-fold CV lift
1	Infertility duration >5 years + No high-quality embryo transferred	43	30	0.059	0.698	1.54	<0.001	0.026	0.555–0.833	1.24–1.86	0.678 ± 0.177	1.50 ± 0.39
2	Age >35 years + BMI ≥24 kg/m² + Day-3/6 embryo transfer	57	38	0.078	0.667	1.47	<0.001	0.026	0.540–0.782	1.19–1.73	0.670 ± 0.100	1.48 ± 0.22
3	Age >35 years + Day-3/6 embryo transfer	91	57	0.124	0.626	1.38	<0.001	0.026	0.525–0.718	1.17–1.60	0.629 ± 0.099	1.39 ± 0.22
4	Age >35 years + BMI ≥24 kg/m² + Pelvic adhesion	48	30	0.065	0.625	1.38	0.010	0.116	0.487–0.750	1.10–1.66	0.602 ± 0.149	1.33 ± 0.33
5	Secondary infertility + Single embryo transferred + Day-3/6 embryo transfer	40	25	0.055	0.625	1.38	0.019	0.122	0.476–0.781	1.06–1.72	0.625 ± 0.117	1.38 ± 0.26
6	Age >35 years + Pelvic adhesion + Day-3/6 embryo transfer	53	33	0.072	0.623	1.37	0.008	0.101	0.492–0.745	1.10–1.65	0.609 ± 0.158	1.35 ± 0.35
7	Age >35 years + Day-3/6 embryo transfer + Gn dose ≥ median	74	46	0.101	0.622	1.37	0.002	0.043	0.508–0.727	1.14–1.60	0.629 ± 0.133	1.39 ± 0.30
8	Age >35 years + Incomplete septate uterus	37	23	0.050	0.622	1.37	0.026	0.122	0.467–0.774	1.02–1.72	0.619 ± 0.158	1.37 ± 0.34
9	Single embryo transferred + Day-3/6 embryo transfer	42	26	0.057	0.619	1.37	0.019	0.122	0.487–0.769	1.08–1.70	0.620 ± 0.135	1.37 ± 0.30
10	Age >35 years + LUFS + Day-3/6 embryo transfer	89	55	0.121	0.618	1.36	<0.001	0.026	0.517–0.712	1.15–1.58	0.622 ± 0.091	1.37 ± 0.20
11	Age >35 years + BMI ≥24 kg/m² + Secondary infertility	88	54	0.120	0.614	1.35	<0.001	0.028	0.516–0.714	1.14–1.57	0.613 ± 0.027	1.35 ± 0.06
12	Age >35 years + BMI ≥24 kg/m² + Infertility duration >5 years	44	27	0.060	0.614	1.35	0.020	0.122	0.465–0.750	1.03–1.66	0.582 ± 0.155	1.28 ± 0.34
13	Infertility duration >5 years + LUFS + ICSI	48	29	0.065	0.604	1.33	0.021	0.122	0.462–0.733	1.02–1.62	0.615 ± 0.200	1.36 ± 0.44
14	Age >35 years + BMI ≥24 kg/m²	90	54	0.123	0.600	1.32	0.002	0.044	0.500–0.700	1.11–1.53	0.599 ± 0.037	1.32 ± 0.08

Rule	Antecedent	n antecedent	n failure	Support	Confidence	Lift	Fisher p	BH-FDR q	Bootstrap 95% CI: confidence	Bootstrap 95% CI: lift	5-fold CV confidence	5-fold CV lift
15	LUFS + ICSI + Day-3/6 embryo transfer	76	45	0.104	0.592	1.31	0.007	0.101	0.487–0.698	1.08–1.54	0.592 ± 0.143	1.31 ± 0.31
16	Infertility duration >5 years + ICSI	49	29	0.067	0.592	1.31	0.031	0.122	0.449–0.724	0.99–1.59	0.599 ± 0.172	1.32 ± 0.38
17	Pelvic adhesion + Day-3/6 embryo transfer + No high-quality embryo transferred	49	29	0.067	0.592	1.31	0.031	0.122	0.454–0.729	1.00–1.61	0.598 ± 0.106	1.32 ± 0.24
18	ICSI + Day-3/6 embryo transfer	78	46	0.106	0.590	1.30	0.007	0.101	0.486–0.697	1.08–1.54	0.589 ± 0.116	1.30 ± 0.25
19	Age >35 years + BMI ≥24 kg/m² + Gn dose ≥ median	73	43	0.100	0.589	1.30	0.010	0.116	0.474–0.703	1.06–1.55	0.587 ± 0.056	1.30 ± 0.13
20	Secondary infertility + Single embryo transferred + No high-quality embryo transferred	51	30	0.070	0.588	1.30	0.031	0.122	0.454–0.714	1.01–1.58	0.587 ± 0.119	1.30 ± 0.27
21	Age >35 years + Infertility duration >5 years + Gn dose ≥ median	58	34	0.079	0.586	1.29	0.024	0.122	0.456–0.703	1.02–1.56	0.597 ± 0.123	1.32 ± 0.27
22	Secondary infertility + ICSI + Day-3/6 embryo transfer	77	45	0.105	0.584	1.29	0.010	0.116	0.479–0.692	1.07–1.53	0.583 ± 0.125	1.29 ± 0.27
23	BMI ≥24 kg/m² + Secondary infertility + No high-quality embryo transferred	60	35	0.082	0.583	1.29	0.024	0.122	0.459–0.717	1.03–1.57	0.570 ± 0.107	1.26 ± 0.24
24	ICSI + Day-3/6 embryo transfer + Gn dose ≥ median	38	22	0.052	0.579	1.28	0.076	0.188	0.437–0.733	0.97–1.62	0.587 ± 0.084	1.30 ± 0.19
25	Secondary infertility + Pelvic adhesion + ICSI	45	26	0.061	0.578	1.28	0.057	0.163	0.434–0.720	0.96–1.60	0.559 ± 0.124	1.23 ± 0.27
26	Day-3/6 embryo transfer + No high-quality embryo transferred	66	38	0.090	0.576	1.27	0.025	0.122	0.453–0.698	1.01–1.54	0.587 ± 0.093	1.30 ± 0.21
27	BMI ≥24 kg/m² + Incomplete septate uterus + Gn dose ≥ median	40	23	0.055	0.575	1.27	0.076	0.188	0.413–0.719	0.93–1.60	0.581 ± 0.184	1.28 ± 0.41
28	Single embryo transferred + No high-quality embryo transferred	54	31	0.074	0.574	1.27	0.043	0.140	0.440–0.700	0.98–1.54	0.569 ± 0.126	1.26 ± 0.28

Rule	Antecedent	n antecedent	n failure	Support	Confidence	Lift	Fisher p	BH-FDR q	Bootstrap 95% CI: confidence	Bootstrap 95% CI: lift	5-fold CV confidence	5-fold CV lift
29	BMI ≥24 kg/m² + No high-quality embryo transferred	61	35	0.083	0.574	1.27	0.033	0.122	0.446–0.698	1.01–1.55	0.560 ± 0.098	1.24 ± 0.22
30	Secondary infertility + Incomplete septate uterus + Day-3/6 embryo transfer	42	24	0.057	0.571	1.26	0.077	0.188	0.417–0.717	0.94–1.59	0.564 ± 0.114	1.25 ± 0.25
31	Incomplete septate uterus + Day-3/6 embryo transfer	44	25	0.060	0.568	1.25	0.077	0.188	0.417–0.710	0.94–1.57	0.557 ± 0.126	1.23 ± 0.28

Table S4. Multivariable logistic regression.

Variable	Adjusted OR	95% CI	p-value
Age, per year	1.04	1.00–1.08	0.031
BMI, per kg/m2	1.00	0.96–1.05	0.860
Infertility duration, per year	0.96	0.92–1.01	0.140
Secondary infertility	2.30	0.95–5.61	0.066
Incomplete septate uterus	0.97	0.61–1.55	0.895
Multiple uterine fibroids	0.93	0.56–1.57	0.798
Bilateral tubal obstruction	1.13	0.80–1.62	0.484
Pelvic adhesion	0.95	0.67–1.33	0.759
LUFS	0.63	0.25–1.61	0.334
Insulin resistance	1.38	0.53–3.60	0.510
Subclinical hypothyroidism	1.20	0.51–2.82	0.675
ICSI vs conventional IVF	1.56	1.02–2.38	0.039
Gn dose, per 1000 IU	1.00	0.81–1.24	0.998
Two embryos transferred vs one	0.55	0.35–0.85	0.008
Day-5 blastocyst transfer vs other	0.49	0.32–0.75	0.001
At least one high-quality embryo transferred	0.63	0.43–0.93	0.020

Table S5. Support/confidence threshold sensitivity.

Analysis	Support threshold	Confidence threshold	Candidate combinations	Rules before pruning	Rules after pruning	FDR q<0.10 rules	Top rule	Top confidence	Top lift
host	0.03	0.5	103	58	49	6	Age >35 years + BMI ≥24 kg/m² + Incomplete septate uterus	0.643	1.42
host	0.03	0.55	103	24	20	4	Age >35 years + BMI ≥24 kg/m² + Incomplete septate uterus	0.643	1.42
host	0.03	0.6	103	11	9	2	Age >35 years + BMI ≥24 kg/m² + Incomplete septate uterus	0.643	1.42
host	0.05	0.5	78	38	32	7	Age >35 years + BMI ≥24 kg/m² + Pelvic adhesion	0.625	1.38
host	0.05	0.55	78	14	11	5	Age >35 years + BMI ≥24 kg/m² + Pelvic adhesion	0.625	1.38
host	0.05	0.6	78	7	5	3	Age >35 years + BMI ≥24 kg/m² + Pelvic adhesion	0.625	1.38
host	0.07	0.5	66	29	24	6	Age >35 years + BMI ≥24 kg/m² + Secondary infertility	0.614	1.35
host	0.07	0.55	66	9	7	4	Age >35 years + BMI ≥24 kg/m² + Secondary infertility	0.614	1.35
host	0.07	0.6	66	3	2	2	Age >35 years + BMI ≥24 kg/m² + Secondary infertility	0.614	1.35
full	0.03	0.5	325	211	183	10	Infertility duration >5 years + Pelvic adhesion + No high-quality embryo transferred	0.731	1.61
full	0.03	0.55	325	98	84	10	Infertility duration >5 years + Pelvic adhesion + No high-quality embryo transferred	0.731	1.61
full	0.03	0.6	325	39	33	8	Infertility duration >5 years + Pelvic adhesion + No high-quality embryo transferred	0.731	1.61
full	0.05	0.5	242	141	121	11	Infertility duration >5 years + No high-quality embryo transferred	0.698	1.54
full	0.05	0.55	242	58	47	9	Infertility duration >5 years + No high-quality embryo transferred	0.698	1.54

Analysis	Support threshold	Confidence threshold	Candidate combinations	Rules before pruning	Rules after pruning	FDR q<0.10 rules	Top rule	Top confidence	Top lift
full	0.05	0.6	242	20	14	7	Infertility duration >5 years + No high-quality embryo transferred	0.698	1.54
full	0.07	0.5	187	101	86	13	Age >35 years + BMI ≥24 kg/m² + Day-3/6 embryo transfer	0.667	1.47
full	0.07	0.55	187	31	25	11	Age >35 years + BMI ≥24 kg/m² + Day-3/6 embryo transfer	0.667	1.47
full	0.07	0.6	187	9	7	7	Age >35 years + BMI ≥24 kg/m² + Day-3/6 embryo transfer	0.667	1.47

Table S6. Age/BMI/infertility duration cutoff sensitivity.

Age cutoff	BMI cutoff	Infertility cutoff	Candidate combinations	Rules after pruning	FDR q<0.10 rules	Top rule	Top n	Top confidence	Top lift	Top q	Age-BMI-pelvic n	Age-BMI-pelvic confidence	Age-BMI-pelvic lift	Age-BMI-pelvic q
35	24	5	78	32	7	Age >35 years + BMI ≥24 kg/m² + Pelvic adhesion	48	0.625	1.38	0.098	48	0.625	1.38	0.098
35	24	7	74	28	7	Age >35 years + BMI ≥24 kg/m² + Pelvic adhesion	48	0.625	1.38	0.093	48	0.625	1.38	0.093
35	24	10	62	22	7	Age >35 years + BMI ≥24 kg/m² + Pelvic adhesion	48	0.625	1.38	0.078	48	0.625	1.38	0.078
35	25	5	74	23	6	Age >35 years + Incomplete septate uterus	37	0.622	1.37	0.213				
35	25	7	72	23	6	Age >35 years + Incomplete septate uterus	37	0.622	1.37	0.207				
35	25	10	60	17	6	Age >35 years + Incomplete septate uterus	37	0.622	1.37	0.173				
35	28	5	63	19	4	Age >35 years + Incomplete septate uterus	37	0.622	1.37	0.246				
35	28	7	60	18	4	Age >35 years + Incomplete septate uterus	37	0.622	1.37	0.237				
35	28	10	51	14	4	Age >35 years + Incomplete septate uterus	37	0.622	1.37	0.202				
38	24	5	61	16	2	Age >35 years	57	0.649	1.43	0.035				
38	24	7	59	15	2	Age >35 years	57	0.649	1.43	0.034				
38	24	10	50	10	2	Age >35 years	57	0.649	1.43	0.029				
38	25	5	59	9	2	Age >35 years	57	0.649	1.43	0.034				
38	25	7	58	11	2	Age >35 years	57	0.649	1.43	0.034				
38	25	10	49	6	2	Age >35 years	57	0.649	1.43	0.028				
38	28	5	51	7	2	Age >35 years	57	0.649	1.43	0.030				
38	28	7	49	8	2	Age >35 years	57	0.649	1.43	0.028				
38	28	10	43	5	2	Age >35 years	57	0.649	1.43	0.025				

Age cutoff	BMI cutoff	Infertility cutoff	Candidate combinations	Rules after pruning	FDR q<0.10 rules	Top rule	Top n	Top confidence	Top lift	Top q	Age-BMI-pelvic n	Age-BMI-pelvic confidence	Age-BMI-pelvic lift	Age-BMI-pelvic q
40	24	5	57	14	0	BMI ≥24 kg/m² + Infertility duration >5 years + Pelvic adhesion	70	0.557	1.23	0.395				
40	24	7	55	13	0	BMI ≥24 kg/m² + Infertility duration >5 years + LUFS	59	0.576	1.27	0.280				
40	24	10	46	8	0	BMI ≥24 kg/m² + Pelvic adhesion + Bilateral tubal obstruction	44	0.545	1.20	0.523				
40	25	5	55	7	0	Infertility duration >5 years + Bilateral tubal obstruction + LUFS	68	0.544	1.20	0.578				
40	25	7	54	9	0	BMI ≥24 kg/m² + Infertility duration >5 years + LUFS	45	0.533	1.18	0.601				
40	25	10	45	4	0	Secondary infertility + Pelvic adhesion + Incomplete septate uterus	50	0.520	1.15	0.775				
40	28	5	47	5	0	Infertility duration >5 years + Bilateral tubal obstruction + LUFS	68	0.544	1.20	0.503				
40	28	7	45	6	0	Secondary infertility + Pelvic adhesion + Incomplete septate uterus	50	0.520	1.15	0.555				
40	28	10	39	3	0	Secondary infertility + Pelvic adhesion + Incomplete septate uterus	50	0.520	1.15	0.639				
